# Pediatric interregional healthcare mobility in Italy

**DOI:** 10.1186/s13052-021-01091-8

**Published:** 2021-06-24

**Authors:** Mario De Curtis, Francesco Bortolan, Davide Diliberto, Leonardo Villani

**Affiliations:** 1 Maternal and Child Health and Urological Sciences Department, University of Rome La Sapienza, Rome, Italy; 2 AGE.NA.S. (AGEnzia NAzionale per i Servizi sanitari regionali), Rome, Italy; 3 Azienda Zero, Regione del Veneto, Padua, Italy; 4 Section of Hygiene, University Department of Life Sciences and Public Health — Università Cattolica del Sacro Cuore, Rome, Italy

**Keywords:** Pediatric population, Healthcare mobility, Healthcare mobility costs, Hospitalizations

## Abstract

**Background:**

The analysis of interregional healthcare mobility represents one of the main criteria for evaluating Regional Healthcare Systems, both in terms of its economic-financial relevance and the quality and satisfaction of the services provided. The aim of the study is to analyze healthcare mobility and its associated cost in Italy in 2019 for all children ≤ 14 years of age.

**Methods:**

We collected data from the “Rapporto annuale sull’attività di ricovero ospedaliero – Dati SDO 2019” published by the Italian Ministry of Health. These data represent the tool for collecting information relating to all hospitalization services provided in accredited public and private hospitals present throughout the national territory. We collected data for all Italian regions and clustered them in two geographical areas: Center-North regions and South regions (including Sicily and Sardinia). We have analyzed the magnitude of the mobility of children among regions and in particular from the South to the Center-North and the relative cost of this interregional mobility.

**Results:**

The hospitalization rate of children residing in the South regions was higher than  that of children residing in the Center-North regions (13.9% vs 12.3%). Children residing in the South were more frequently treated in other regions than those living in the Center-North (11.9% vs 6.9%). Even considering the high complexity hospitalizations, children living in the South more frequently underwent treatment in other regions (21.3% vs 10.5% of the Center-North). The cost of passive mobility amounts to € 103.9 million for the South regions (15.1% of the total hospitalizations’ expenditure) and the 87.1% of this cost refers to the mobility to the hospitals of Center-North. The cost of healthcare migration from South regions to other South regions was much lower (12.9%, equal to € 13.4 million).

**Conclusions:**

Healthcare mobility, while affecting all Italian regions, is particularly relevant in the South regions and indicates a lack of pediatric care, which should be strengthened by creating services that are currently not evenly distributed throughout the territory.

## Background

In Italy, the National Health Service (NHS, Servizio Sanitario Nazionale – SSN) guarantees universal care. In particular, the Italian NHS is a “three layers” public universal healthcare system, free of charge at the point of care [1, 2]. At national level, the Ministry of Health defines the healthcare principles and priorities, through the identification of the core benefit package of services (Livelli Essenziali di Assistenza – LEA) guaranteed across the whole country, based on collective prevention and public health, primary care and hospital care [3, 4]. Moreover, the Ministry of Health allocates funds to the regions in order to provide healthcare services and it monitors the activities of the regions. Italian regions (19 regions and 2 Autonomous Provinces), indeed, are responsible for ensuring the organization and the delivery of services through their Regional Healthcare Systems, according to the LEA defined at national level [4]. At local level, the provision of services is ensured by a regional network of Local Health Units (Azienda Sanitaria Locale – ASL) and autonomous public and private hospitals. In this context, each citizen refers to the Regional Healthcare System in which he resides. However, citizens can decide whether to be assisted by thier Regional Healthcare System or in other regions, defining the concept of active and passive interregional mobility [5]. This concept represents, at the national level, a phenomenon of healthcare mobility already known at the international level [6–8]. In Italy, since there are 21 different healthcare systems, it is possible to observe, therefore, the same phenomenon, albeit in a national context. In particular, active mobility indicates the attraction index of a region and identifies the healthcare services offered to non-resident citizens, while passive mobility identifies the healthcare services provided to citizens outside the region of residence (also known as escape index) [9]. Therefore, the attraction index measures the capacity of a region to attract patients from other regions. It is calculated as the proportion between the number of  hospital discharges of non-resident patients in a region and the total number of hospitalizations carried out in that region. The escape index, on the contrary, quantifies the propensity of the patients to move away from their own region in order to take advantage of a healthcare service. It is calculated as the proportion between the number of hospital discharges of patients residing in a region and the total number of hospitalizations of residents in that region across the national territory. In both cases, foreign citizens and those of unknown nationality are excluded [9].

Factors such as effectiveness and efficiency of each Regional Healthcare Service, presence of Reference Centers for specific diseases, waiting lists, diagnostic services and availability of treatments, and perceived or real quality of assistance, might influence patients’ mobility [10].

Therefore, both the active and passive mobility are indicators that could be interpreted as a proxy measure of the quality (real or perceived) of the healthcare assistance provided in a region. In particular, a high index of escape may be due to deficiencies in the supply of care, while a high index of attraction may be due to a higher quality of healthcare assistance in a specific region [5, 9].

From an economic point of view, active mobility represents a credit item for the regions, while passive mobility represents a debt item; each year the region that provides the service is reimbursed by that of the citizen’s residence [11, 12].

The economic value of interregional healthcare mobility for the entire Italian population in 2018 (hospitalizations, outpatient specialists, basic medicine, territorial pharmaceuticals and direct administration of drugs and healthcare transport) was equal to a limited percentage (4.1%) of total healthcare expenditure (approximately € 113 billion). However, it has particular importance due to the impact on the financial balance (positive or negative) of regions (regions with a balance > € 100 million: Lombardy + € 750 million, Emilia-Romagna + € 327 million, Tuscany + € 144 million, Veneto + € 139 million; regions with balance < − € 200 million: Puglia - € 211 million, Sicily - € 223 million, Calabria - € 288 million, Campania - € 351 million) [5]. The analysis of healthcare mobility, therefore, represents one of the main performance indicators of Regional Healthcare Systems, both for its economic relevance and for the adequacy / satisfaction of the services provided [12].

While the data of healthcare mobility of the entire population are known [9], there is lack of evidence about both active and passive healthcare mobility of children ≤14 years old. In particular, since children living in the South regions are the most disadvantaged from an economic, educational and social point of view [13], we focused on the healthcare mobility from the South regions to the Center-North regions. In this context, the aim of the study is to analyze the healthcare mobility in 2019 in Italy for all children ≤ 14 years of age and its associated cost.

## Methods

We collected data from the “Rapporto annuale sull’attività di ricovero ospedaliero – Dati SDO 2019” published by the Italian Ministry of Health in collaboration with AGENAS (AGEnzia NAzionale per i Servizi sanitari regionali) [9]. The report includes all the hospital discharge forms (Scheda Dimissione Ospedaliera – SDO), collecting information on all inpatient services provided in public and private accredited hospitals throughout the national territory. The hospitalization services provided in 2019 to patients 0–14 age at all accredited public and private structures in the country were considered and, at the same time, those identified as ‘highly complex’ by the “Interregional Agreement for the compensation of Health Mobility year 2019” [11].

Regions and Autonomous Provinces have been divided into two groups, according to their geographical location: Center-North regions (Piedmont, Valle d’Aosta, Lombardy, Bolzano, Trento, Veneto, Friuli Venezia Giulia, Liguria, Emilia-Romagna, Tuscany, Umbria, Marche and Lazio) and South regions (Abruzzo, Molise, Campania, Puglia, Basilicata, Calabria, Sicily and Sardinia).

We evaluated the burden of the active and passive mobility for children ≤ 14 years of age, considering all the Diagnosis Related Group (DRG), including the high complexity DRG. In particular, for each region and macroarea we calculated the escape index on the total hospitalizations and the related costs.

Finally, we analyze the impact of the mobility of children between the different areas of Italy, focusing on the mobility from South regions to the Center-North. The children population in 2019 was obtained from the National Institute of Statistics (ISTAT) [14].

## Results

In Italy, in 2019 there were 1,009,904 hospitalizations among the population ≤ 14 years old, of which 921,491 (91.2%) were within the region of residence. While homogeneous values are observed considering the volumes of hospitalization on the basis of resident population, with variations from a minimum value of 10.8% in Veneto to a maximum of 15.1% in Lazio, there are important differences between Center-North and South regions. In particular, a higher rate of hospitalization was observed among children of South regions (13.9% vs 12.3**%).** The mean per-capita cost of hospitalization in children of South and Center-North regions was € 252 and € 208, respectively.

Moreover, the escape index on the total hospitalizations varies enormously, from 4.2% in Lazio to 40.1% in Molise (Table 1). The overall expenditure for hospitalizations has been equal to approximately € 1.7 billion, of which € 193.6 million refer to passive mobility. Of this, more than 50% is sustained by the 8 regions of the South (€ 103.9 million). The mean escape index costs’ in the South regions is equal to 15.1%, almost double that of the Center-North (8.4%) (Table 2).

**Table 1:** Hospitalizations and escape index in Italy in the 0–14 population (2019)

Region	Population 0–14	Total hospitalizations	Hospitalization rate (%)	In-region hospitalizations	Out-region hospitalizations	Escape index (%)
**North and Center**	**5,146,568**	**632,027**	**12.3**	**588,586**	**43,441**	**6.9**
Piedmont	535,335	60,640	11.3	55,509	5131	8.5
Valle d’Aosta	16,475	1803	10.9	1356	447	24.8
Liguria	170,174	24,998	14.7	22,695	2303	9.2
Lombardy	1,362,146	162,115	11.9	154,320	7795	4.8
Trento	77,874	9030	11.6	7613	1417	15.7
Bolzano	83,353	10,243	12.3	9676	567	5.5
Veneto	646,974	69,553	10.8	64,546	5007	7.2
Friuli Venezia Giulia	145,473	16,064	11.0	14,824	1240	7.7
Emilia-Romagna	583,135	67,025	11.5	62,093	4932	7.4
Tuscany	457,615	57,279	12.5	54,537	2742	4.8
Umbria	108,760	13,512	12.4	10,066	3446	25.5
Marche	191,725	24,220	12.6	20,603	3617	14.9
Lazio	767,529	115,545	15.1	110,748	4797	4.2
**South**	**2,725,319**	**377,877**	**13,9**	**332,905**	**44,972**	**11.9**
Abruzzo	161,223	23,103	14.3	17,996	5107	22.1
Molise	34,355	4589	13.4	2747	1842	40.1
Campania	832,055	121,948	14.7	110,401	11,547	9.5
Puglia	520,560	71,312	13.7	63,539	7773	10.9
Basilicata	66,379	8873	13.4	6311	2562	28.9
Calabria	252,792	35,787	14.2	28,324	7463	20.9
Sicily	676,331	89,779	13.3	83,187	6592	7.3
Sardinia	181,624	22,486	12.4	20,400	2086	9.3
**Italy**	**7,871,887**	**1,009,904**	**12.8**	**921,491**	**88,413**	**8.8**

**Table 2:** Overall costs of hospitalizations and passive mobility costs in Italy (2019)

Region	Population 0–14	Total cost of hospitalizations (million of €)	In-region hospitalizations’ costs (million of €)	Out-region hospitalizations’ costs (million of €)	Costs of passive mobility/total costs (%)
**North and Center**	**5,146,568**	**1070.6**	**980.9**	**89.7**	**8.4**
Piedmont	535,335	120.5	110.3	10.2	8.5
Valle d’Aosta	16,475	3.5	2.3	1.2	34.3
Liguria	170,174	44.5	40.1	4.4	9.9
Lombardy	1,362,146	272.4	255.4	17.0	6.2
Trento	77,874	15.3	11.8	3.5	22.9
Bolzano	83,353	14.0	12.1	1.9	13.6
Veneto	646,974	126.0	116.3	9.7	7.7
Friuli Venezia Giulia	145,473	26.3	22.8	3.5	13.3
Emilia-Romagna	583,135	122.9	112.7	10.2	8.3
Tuscany	457,615	96.2	90.6	5.6	5.8
Umbria	108,760	24.5	18.3	6.2	25.3
Marche	191,725	41.2	33.4	7.8	18.9
Lazio	767,529	163.3	154.8	8.5	5.2
**South**	**2,725,319**	**688.5**	**584.5**	**103.9**	**15.1**
Abruzzo	161,223	39.5	29.1	10.4	26.3
Molise	34,355	7.4	4.0	3.4	45.9
Campania	832,055	211.8	186.3	25.5	12.0
Puglia	520,560	134.6	116.2	18.4	13.7
Basilicata	66,379	15.4	8.6	6.8	44.2
Calabria	252,792	63.8	46.7	17.1	26.8
Sicily	676,331	176.5	159.7	16.8	9.5
Sardinia	181,624	39.5	33.9	5.6	14.2
**Italy**	**7,871,887**	**1759.2**	**1565.6**	**193.6**	**11.0**

As shown in Table 3, analyzing the flows between regions, important differences are observed. In the Center-North, in fact, 43,441 hospitalizations were performed outside the region of residence (6.9% of the total). Of these, however, 37,884 (87.2%) are carried out in other regions of the Center-North, while 5557 (12.8%) are carried in the South regions. This mobility corresponds to an expenditure of € 89.7 million, of which € 80.7 million (90%) involve the mobility to Center-North.

**Table 3:** Passive mobility and related costs between geographical areas of Italy (2019)

Area of residence	Population 0–14	Total hospitalizations (% on the total population) and related costs (total and per-capita)	Out-region hospitalizations (%) and related costs	Passive mobility to regions of Center-North (%) and related costs	Passive mobility to regions of South (%) and related costs
Center-North	5,146,568	632,027 (12.3%)	43,441 (6.9%)	37,884 (87.2%)	5557 (12.8%)
		€ 1070 million (€ 208 per-capita)	€ 89.7 million (8.4%)	€ 80.7 million (90%)	€ 9.0 million (10%)
South	2,725,319	377,877 (13.9%)	44,972 (11.9%)	38,462 (85.5%)	6510 (14.5%)
		€ 688 million (€ 252 per-capita)	€ 103.9 million (15.1%)	€ 90.5 million (87.1%)	€ 13.4 million (12.9%)

On the contrary, in the South regions there were 44,972 out-region hospitalizations (equal to 11.9% of the total, a value almost double that of the Center-North), of which 38,462 (85.5%) are carried out in regions of the Center-North. Only 14.5% are carried out in other regions of the South (Table 3). This mobility corresponds to a total expenditure of € 103.9 million euros, of which 87.1% (€ 90.5 million) involves the mobility to Center-North.

The phenomenon is even more amplified considering “high complexity” hospitalizations (Table 4). Out of 16,673 hospitalizations in the Center-North, in fact, 1758 (10.5%) are carried out in other regions. Of these, 1673 (95.2%) are carried out in other regions of the Center-North, while 85 (4.8%) are carried in regions of the South. This mobility corresponds to a total expenditure of € 29.5 million, of which 93.6% involves the mobility to other regions of Center-North.

**Table 4:** Passive mobility and related costs between geographical areas of Italy for high complexity hospitalizations (2019)

Area of residence	Population 0–14	Total high complexity hospitalizations (% on the total population) and related costs (total and per-capita)	Out-region hospitalizations (%) and related costs	Passive mobility to regions of Center-North and related costs	Passive mobility to regions of South and related costs
Center-North	5,146,568	16,673 (3.3%)	1758 (10.5%)	1673 (95.2%)	85 (4,8%)
		€ 302 million (€ 59 per-capita)	€ 29.5 million (9.7%)	€ 27.6 million (93.6%)	€ 1.9 million (6.4%)
South	2,725,319	9978 (3.7%)	2127 (21.3%)	1867 (87.8%)	260 (12.2%)
		€ 186 million (€ 68 per-capita)	€ 36.6 million (19.7%)	€ 32.3 million (88.3%)	€ 4.3 million (11.7%)

On the contrary, in the South regions, out of 9978 high complexity hospitalizations, 2127 (21.3%) are carried out in other regions (more than double of the Center-North). Of these, 1867 (87.8%) are carried out in Center-North regions. This data corresponds to a mobilization of resources towards the regions of Center-North amounting to € 32.3 million, on a total cost for high complexity hospitalizations of € 186 million (17.4%).

**Fig. 1:**
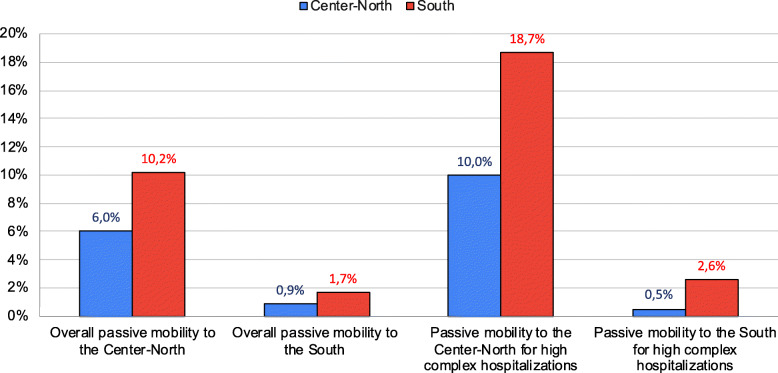
Passive mobility between geographical areas in Italy: hospitalizations and high complexity hospitalizations (2019)

**Fig. 2:**
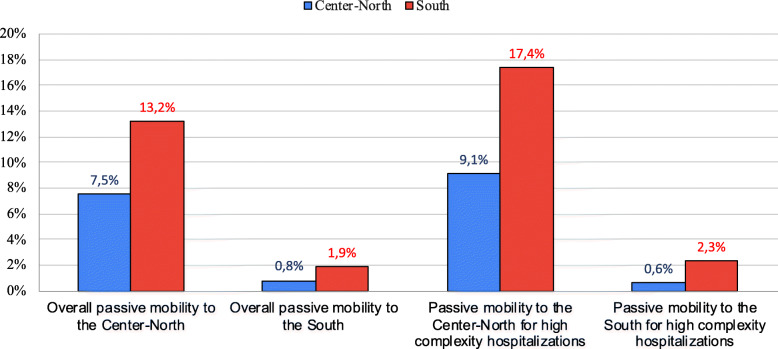
Costs (percentage on the total cost of hospitalizations) of passive mobility between geographical areas in Italy for overall hospitalizations and high complexity hospitalizations (2019)

From the above tables, partially summarized in Figs. 1 and 2, it can be seen that that escape index and associated costs are amplified when we limit the observation to high complexity hospitalizations only; a significant portion of the outflow to South regions is received by the facilities of the Lazio region.

In all regions and Autonomous Provinces, even those provided of specific mono-specialized pediatric facilities, it is evident a not negligible part of out-region mobility, which often approaches 10%. This phenomenon, at least in part due to the hyperspecialization of skills, leads patients to research the Centers according to their reputation or to the uniqueness of the healthcare assistance (i.e., diagnostic services and availability of treatments). Especially patients who need high complexity services show a general greater propensity to move outside region, as these performances are provided in a limited number of structures, with the need of significant resources and specialized skills.

## Discussion

In this study we evaluated for the first time the escape index for children ≤ 14 years old across Italian regions. The number of hospitalizations and healthcare mobility from the region of residence to another are both important indicators to evaluate the adequacy of the regional healthcare service and proper healthcare planning.

Our study confirms the critical situation of pediatric care in some regions and especially in the South regions. In particular, children residing in the South regions compared with those residing in the Center-North are more frequently hospitalized and more frequently transferred to other regions for healthcare assistance. The magnitude of transfers from the South to the Center-North regions has a very high cost for some regions. In Calabria, for example, it exceeds a quarter of all expenditure for healthcare assistance. Moreover, the escape index of patients from the Center-North is often (87.2%) directed towards other regions in the same area (proximity mobility). On the contrary, patients from the South tend to move mainly towards regions in the Center-North (85.9%).

The entity of this mobility is even greater considering the ‘high complexity’ hospitalizations: it occurs, indeed, in 21.3% of cases in the South regions, of which 87.8% involves the Center-North.

Therefore, patients living in the South recourse much more to the interregional healthcare mobility (especially for high complexity pathologies), with a particular propensity to the healthcare facilities of the Center-North, even if geographically distant. This mobility highlights the need for an expansion/improvement of services related to pediatric care in the South. However, it should also be noted that there are good healthcare facilities in the South, and, with a patients’ correct information, many transfers (especially those not of high complexity) could be reduced.

Greater attention needs to be given to children and young people living in the South regions, who are known to be at greater risk than those born in the Center-North as they have a higher poverty index and a higher infant mortality rate [13, 15].

In addition, regions in the South registered a lower public health expenditure pro-capita than regions in the Center-North in the last 20 years [16, 17], in part due to the Recovery Plans of economic deficits that have led to a significant reduction in funding for healthcare [18]. In fact, as of March 2021, six of the seven regions subject to the discipline of the deficit Recovery Plans are in the South [19].

The healthcare mobility of children determines profound suffering due to the detachment from their place of origin, economic problems for families due to the overall cost of the transfer and work difficulties for parents. In addition, the South regions, for this healthcare mobility, are forced to reimburse, through the mechanism of compensation between regions, the healthcare services provided to their own citizens in other regions [11]. This cost, which was more than € 90 million in 2019, could instead partially be invested locally to improve healthcare assistance.

The high level of passive mobility from Southern regions to hospitals in Center-North stems, at least in part, from the lack of highly specialized hospitals in the South. However, high complexity hospitalizations represent only about 2.5% of the total. Therefore, in order to reduce passive mobility, it would be necessary on the one hand to treat locally non-high complexity diseases by promoting an improvement in the quality of hospitals in the southern regions impacted by budget cuts in recent years, and on the other hand to create collaborations, including through innovative digital tools (e.g., telemedicine), with hospitals in Center-North Italy, to ensure quality healthcare for the management of patients with high complexity diseases.

## Conclusions

The funds provided by the Next Generation EU represent a unique and perhaps unrepeatable opportunity for the restart of our country [20]. It is essential to correct the serious gap between the regions and improve the organization of the healthcare system and the education system, starting from the almost non-existent kindergartens in the South regions to the universities.

Only by contrasting economic and educational poverty and the difficult healthcare situation in the South, Italy will be able to break down the profound inequalities that afflict the country. The investment in childhood is the most effective, the most lasting and the best contribution to economic recovery and to the development of a society.

## Data Availability

All data were collected from National and Regional databases.

## Abbreviations

NHS: National Health Service
SSN: Servizio Sanitario Nazionale
LEA: Livelli Essenziali di Assistenza
ASL: Azienda Sanitaria Locale
AGENAS: AGEnzia NAzionale per i Servizi sanitari regionali
SDO: Scheda Dimissione Ospedaliera
DRG: Diagnosis Related Group
ISTAT: National Institute of Statistics
